# A validation of diagnostic score for molecular analysis of hereditary autoinflammatory syndromes with periodic fever in Turkish children

**DOI:** 10.1186/1546-0096-9-S1-P298

**Published:** 2011-09-14

**Authors:** Erkan Demirkaya, Cengizhan Acikel, Isabella Ceccherini, Seza Özen, Abdulbaki Karaoglu, Ismail Dursun, Betul Sozeri, Yelda Bilginer, Zubeyde Gunduz, Yusuf Tunca, Katerina Oikonomaki, Ioanna Varela, Francesco Caroli, Maria Pia Sormani, Marco Gattorno

**Affiliations:** 1 Gulhane Military Medical Academy, Pediatric Nephrology and Rheumatology Unit, Ankara, Turkey; 2 Gulhane Military Medical Faculty, Department of Public Health, Division of Epidemiology, Ankara, Turkey; 3 Laboratorio di Genetica Molecolare, Istituto Giannina Gaslini, Genova, Italy; 4 Hacettepe University Faculty of Medicine, Department of Pediatrics, Pediatric Nephrology and Rheumatology Unit, Ankara, Turkey; 5 Kayseri Education and Research Hospital, Kayseri,Turkey; 6 Ege University Faculty of Medicine Department of Pediatrics, Division of Nephrology, Izmir, Turkey; 7 Erciyes University Medical School, Pediatric Nephrology & Rheumatology Unit, Kayseri, Turkey; 8 Gulhane Military Medical Academy, Department of Medical Genetics, Ankara, Turkey; 9 University of Genoa, Genoa, Italy; 10 Giannina Gaslini Institute, 2nd Division of Paediatrics, Genoa, Italy

## Objective

In the present study, we validated a set of variables that predict the presence of a mutation in one of the 3 genes known to be associated with the common monogenic autoinflammatory diseases.

## Methods

A total of 93 consecutive patients with a clinical history of periodic fever were screened for mutations in the *MVK*, *TNFRSF1A*, and *MEFV* genes, and detailed clinical information was collected prospectively. For autosomal-recessive diseases (MKD and FMF), only homozygous or compound heterozygous patients were accepted as genetically positive. The developed diagnostic score was validated in an independent set of 93 patients.

## Results

93 patients whose suspected diagnosis was one of the autoinflammatory diseases were enrolled. The mean age at onset was 58,64+40,69 months (54 F, 39 M;). A total of 25 patients with periodic or recurrent fevers did not display any mutation in the 3 genes examined. Of these 68 patients with positive findings on genetic testing, 30 of them had a mutation only in one allele and 38 had mutations in two alleles for MEFV gene. In addition, we identified 4 low-penetrance R92Q mutations for TRAPS and 3 patients, who were compound heterozygotes for the MVK gene. The diagnostic score revealed moderate sensitivity (68%) and specificity (58%) for discriminating patients who were genetically positive from those who were negative.

## Conclusion

The suggested set of variables may be used to decide on genetic screening for patients with periodic fever syndromes. However, further studies are needed to improve the sensitivity of the suggested score.

